# *De novo* whole-genome assembly of the *Aeromonas veronii* strains KMM289, KMM358, and KMM366 isolated from threespine stickleback (*Gasterosteus aculeatus*) in Alaska, USA

**DOI:** 10.1128/mra.00841-26

**Published:** 2026-09-21

**Authors:** Sihan Bu, Nataly Vargas, Sarah Pasqualetti, Caroline Vieira Da Silva, Emily Lescak, Kat Milligan-McClellan

**Affiliations:** 1 Department of Molecular and Cell Biology, University of Connecticut 7712, Storrs, Connecticut, USA; 2 Department of Biological Sciences, University of Alaska Anchorage 3291, Anchorage, Alaska, USA; 3 International Arctic Research Center, University of Alaska Fairbanks 11414, Fairbanks, Alaska, USA; University of Manitoba, Winnipeg, Canada

**Keywords:** *de novo* whole-genome assembly, *Aeromonas veronii*, threespine stickleback, *Gasterosteus aculeatus*

## Abstract

We report the genome sequences of the *Aeromonas veronii* strains KMM289, KMM358, and KMM366 isolated from one freshwater and two anadromous threespine stickleback fish (*Gasterosteus aculeatus*) in Alaska, USA. Genomes were assembled by a hybrid *de novo* approach with Oxford Nanopore long-read and Illumina short-read sequencing data.

## ANNOUNCEMENT

*Aeromonas veronii* is a gram-negative bacterium found in aquatic environments and associated with infections in aquatic organisms (1, 2). Its persistence across diverse ecological niches highlights bacterial adaptation to selective pressures encountered in both free-living and host-associated environments, which may contribute to strain-level variation. In this study, strain KMM289 was isolated from freshwater threespine stickleback fish (*Gasterosteus aculeatus*) collected from Cheney Lake (61°12′01.1″N, 149°45′46.0″W) in 2016, and strains KMM358 and KMM366 were isolated from anadromous stickleback captured from Rabbit Slough (61°32′13.9″N, 149°15′11.9″W) in 2022; both sites are located in Alaska, USA. These strains were selected to examine whether host population variation is associated with microbial phenotypic and genotypic variation.

Microbes from dissected fish guts were isolated as described in reference 3. The taxonomy of the strains was confirmed by V1-V9 16S ribosomal RNA (rRNA) gene sequencing analysis through Sanger sequencing, as described in reference 4, using bacterial cell pellets submitted to GeneWiz (New Jersey, USA). For whole-genome sequencing, strains were streaked onto Luria-Bertani (LB) agar from freezer stocks and incubated at 25°C overnight. Pure colonies were inoculated in 5 mL of LB medium and incubated at 25°C at 200 rpm overnight. Genomic DNA was extracted using the DNeasy Blood and Tissue Kit (Qiagen, Germany) according to the manufacturer’s instructions. DNA was sent to Plasmidsaurus (South San Francisco, USA) for library preparation and hybrid whole-genome sequencing. Oxford Nanopore Technologies (ONT) libraries were prepared using the Rapid Barcoding Kit 96 V14 (SQK-RBK114.96), and sequencing was performed on PromethION P24 with R10.4.1 flow cells. Illumina libraries were prepared using the Illumina DNA Prep Kit (20060059), and sequencing was performed on NovaSeq X Plus (paired-end 2 × 150 bp reads).

All bioinformatic analyses were performed with default settings. The quality control for raw read data was performed using FastQC (v0.12.1) (5) for short reads and NanoPlot (v1.41.6) (6) for long reads. For KMM366, short reads were deduplicated using BBMap (v39.08) (7) as an additional step as FastQC indicated extensive duplication. Short reads were filtered using fastp (v0.23.2) (8) and subsequently trimmed using Trimmomatic (v0.39) (9). Long reads were filtered using Porechop (v0.2.4) (10) and then trimmed using NanoFilt (v2.6.0) (6). Sequence data were assembled using Unicycler (v0.5.0) (11) for a hybrid *de novo* assembly. Plasmids were detected using MOB-suite (v3.1.9) (12), which identified one KMM358 contig and two KMM366 contigs as plasmid-derived sequences. Based on the above analyses, KMM289 and KMM366 were draft genomes, whereas KMM358 was resolved as a circular complete genome assembly. Genome quality, completeness, and contamination were assessed using QUAST (v5.2.0) (13), CheckM (v1.1.3) (14), and BUSCO (v6.0.0) (15) with the aeromonas_odb12 data set.

Genome size ranged from 4.74 to 4.91Mb with 58.20 to 58.59% GC content. BUSCO completeness was 99.3 to 99.6% with few missing BUSCOs, indicating high recovery of expected gene contents. CheckM estimated genome completeness above 99% with contamination values of 0.13 to 0.93%. Genome annotation was performed by the National Center for Biotechnology Information (NCBI) Prokaryotic Genome Annotation Pipeline (v6.11) upon NCBI submission (16). The characteristics of the assembled genomes are summarized in Table 1.

**TABLE 1:** Key characteristics of the *Aeromonas veronii* strains KMM289, KMM358, and KMM366

Characteristics	KMM289	KMM358	KMM366
Raw reads data			
Illumina sequencing			
Read pairs	1,529,005	1,465,808	22,523,637
Average sequence length (bp)	159	159	159
Total bases (nt)	486,223,590	466,126,944	7,162,516,566
SRA acc. no.	SRR38464981	SRR38408131	SRR38427396
ONT sequencing			
Read count	137,044	63,130	89,304
Average sequence length (bp)	6,526.8	3,961.5	5,491.2
Total bases (nt)	894,460,053	250,090,847	490,386,450
SRA acc. no.	SRR38429962	SRR38429942	SRR38427570
Genome data			
Assembly status	Draft	Complete	Draft
Genome coverage^*a*^	291×	148×	1,558×
Genome acc. no.	JCAFXH000000000	JBYHEI000000000	JBYHEJ000000000
Total length (Mb)	4.74	4.85	4.91
Number of contigs	1	2	4
GC content (%)	58.59	58.32	58.20
N50 (Mb)	4.74	4.74	4.75
CheckM completeness (%)	99.46	99.68	99.68
Contamination (%)	0.13	0.89	0.93
BUSCO completeness (%)	99.3	99.6	99.6
Missing BUSCOs	13	5	5

^*a*^ Genome coverage was calculated by dividing the total number of bases generated from Illumina and ONT sequencing by the final assembly size.

## Data Availability

The genomic data of the *Aeromonas veronii* strains KMM289, KMM358, and KMM366 have been deposited at NCBI GenBank under project number PRJNA1462700, PRJNA1462770, and PRJNA1463156, respectively. Raw reads and assemblies are available under the accession numbers reported in Table 1. The 16S rRNA gene sequences of strains are available in the NCBI GenBank under accession numbers PX519571, PX519590, and PX519598, respectively.
